# Decoding of Self-paced Lower-Limb Movement Intention: A Case Study on the Influence Factors

**DOI:** 10.3389/fnhum.2017.00560

**Published:** 2017-11-23

**Authors:** Dong Liu, Weihai Chen, Ricardo Chavarriaga, Zhongcai Pei, José del R. Millán

**Affiliations:** ^1^School of Automation Science and Electrical Engineering, Beihang University, Beijing, China; ^2^Defitech Chair in Brain-Machine Interface, École Polytechnique Fédérale de Lausanne, Geneva, Switzerland

**Keywords:** brain-machine interface (BMI), electroencephalography (EEG), lower-limb movement, onset detection, movement-related cortical potentials (MRCPs), sensory motor rhythms (SMRs)

## Abstract

Brain-machine interfaces (BMIs) have been applied as new rehabilitation tools for motor disabled individuals. Active involvement of cerebral activity has been shown to enhance neuroplasticity and thus to restore mobility. Various studies have focused on the detection of upper-limb movement intention, while the fewer study has investigated the lower-limb movement intention decoding. This study presents a BMI to decode the self-paced lower-limb movement intention, with 10 healthy subjects participating in the experiment. We varied four influence factors including the movement type (dorsiflexion or plantar flexion), the limb side (left or right leg), the processing method (time-series analysis based on MRCP, i.e., movement-related cortical potential or frequency-domain estimation based on SMR, i.e., sensory motor rhythm) and the frequency band (e.g., delta, theta, mu, beta and MRCP band at [0.1 1] Hz), to estimate both single-trial and sample-based performance. Feature analysis was then conducted to show the discriminant power (DP) and brain modulations. The average detection latency was −0.334 ± 0.216 s in single-trial basis across all conditions. An average area under the curve (AUC) of 91.0 ± 3.5% and 68.2 ± 4.6% was obtained for the MRCP-based and SMR-based method in the classification, respectively. The best performance was yielded from plantar flexion with left leg using time-series analysis on the MRCP band. The feature analysis indicated a cross-subject consistency of DP with the MRCP-based method and subject-specific variance of DP with the SMR-based method. The results presented here might be further exploited in a rehabilitation scenario. The comprehensive factor analysis might be used to shed light on the design of an effective brain switch to trigger external robotic devices.

## 1. Introduction

A brain-machine interface (BMI), also known as a brain-computer interface (BCI), is a communication and control system that does not require any peripheral muscular activity (Wolpaw et al., 2002). The brain activity is translated into control signals bypassing the physiological output pathways, thereby enabling severely disabled individuals to interact with the surroundings. There have been assistive BMIs for communication in paralysis patients with amyotrophic lateral sclerosis and rehabilitative BMIs for end-users with chronic stroke (Naseer et al., 2014; Chaudhary et al., 2016). Furthermore, many studies have demonstrated that active involvement of central nervous system can improve neuroplasticity and thus enhance the opportunity of motor recovery (Beldalois et al., 2011; Hatem et al., 2016). In this respect, non-invasive EEG-based BMI has been developed to decode the user's movement intention based on markers of active brain involvement in the preparation of the desired movement.

Current state-of-the-art BMIs have employed two types of EEG correlates to detect motor intention, i.e., movement-related cortical potentials (MRCPs) and sensory motor rhythms (SMRs). MRCPs are slow EEG fluctuations associated with movement planning and execution, which occur as early as 1.5 s to 2 s before the actual movement onset (Kornhuber and Deecke, 1965; Libet, 1993). Recent works have used MRCPs to identify self-paced reaching movement intention in a single-trial basis (Lew et al., 2012) and detect both motor imagery and motor execution of ankle dorsiflexion in real time (Xu et al., 2014a). MRCPs have also been used to explore the differences between goal-directed and non-goal-directed movements and the classification results showed that better performance would be achieved when the movement was directed toward a goal (Pereira et al., 2017). SMRs, on the other hand, have also been used as an alternative to MRCPs for movement intent decoding. Typical SMRs include event-related desynchronization/synchronization (ERD/ERS), which refer to the decrease and increase of power in given frequency intervals, e.g., mu (8–12 Hz) and beta bands (13–30 Hz) (Pfurtscheller and Da Silva, 1999). For instance, self-paced wrist movement onset was detected from ERD-based EEG correlates from healthy subjects (Bai et al., 2011). Another work by Ibáńez et al. also used self-paced wrist extension as the movement type to detect the motor intention from essential tremor patients (Ibáńez et al., 2013). Besides, post-imagery ERS, known as beta rebound, has also been investigated to detect movement intention (Pfurtscheller and Solis-Escalante, 2009). The ERS is observed after the movement onset with several seconds delay, which hardly satisfies the underlying Hebbian principle for intent detection (Xu et al., 2014a). Consequently, in this work, SMR-based intent detection only refers to the power decrease or ERD.

More recently, some BMIs have combined MRCPs and SMRs in order to boost their decoding performance, as both of the EEG correlates have been observed to provide complementary information regarding the timing of volitional motor actions. Voluntary upper-limb reaching movement onset was detected with a logistic regression classifier combining the output of an SMR-based naive Bayes classifier and an MRCP-based matched filter (Ibáńez et al., 2014). Furthermore, movement types and movement directions have been detected from pre-movement EEG correlates. Stand-up and sit-down transitions were classified in (Bulea et al., 2014) with both externally triggered and self-paced paradigms. Another study by Lew et al. shows that reaching directions can be classified from MRCPs preceding movement onset in a self-paced paradigm (Lew et al., 2014). Passive and active center-out reaching movement decoding were further investigated in Úbeda et al. (2017) and results showed that the low-frequency bands carried most of the significant information for the kinematics decoding

Previous studies have mainly relied on upper-limb movement intention to build a brain switch and results indicated that the decoding performance was influenced by movement types, frequency bands and processing techniques, e.g., MRCP-based and SMR-based methods. While upper-limb movement intention has been shown to provide multifaceted and rich information as control signals, several works have been done to tackle the lower-limb movement decoding. Locomotor training was evaluated with stroke patients and the effectiveness was estimated comparing with home-exercise in Duncan et al. (2011) and physiotherapy in Pohl et al. (2007). Body weight supported treadmill training and conventional over-ground walking training were further compared in Mao et al. (2015) and a review of the techniques and therapies used in gait rehabilitation after stroke can be found in Beldalois et al. (2011). Besides, lower-limb motor intention can be used to build an intuitive and natural BMI to trigger gait-related movements. A recent study has shown that it was possible to decode walking intention from cortical patterns generated in the sensorimotor strip during robot-assisted gait training in both healthy volunteers and stroke patients (Garcia-Cossio et al., 2015).

For lower-limb motor intent detection, the most commonly employed movement was ankle dorsiflexion of the dominant leg (Niazi et al., 2011; Xu et al., 2014a), although there have been also attempts to decode plantar flexion (Boye et al., 2008). Dorsiflexion refers to flexion between the foot and the body's dorsal surface, where the toes are brought closer to the shin. In contrast to dorsiflexion, plantar flexion refers to the movement where the angle between the foot and the body's plantar surface decreases during the movement. In contrast to the works by Xu et al. where a motorized unilateral orthosis was used to conduct the movement (Xu et al., 2014b), in this study we performed the experiment with a lower-limb gait trainer (Liu et al., 2017). Consequently, limb side, i.e., moving left or right leg, was considered as another influence factor for the intention decoding.

Therefore, the motivation of this study is twofold. First, to build a brain switch based on the decoding of self-paced lower-limb movement intention. Second, to address the gap in the literature with the analysis of influence factors on the performance. In this work, we conducted experiment with 10 healthy individuals and compared the detection of the movement intention with respect to four influence factors: movement types (ankle dorsiflexion and plantarflexion), limb sides (left and right legs), processing frameworks (MRCP-based method in the time domain and SMR-based method in the frequency domain) and frequency bands (e.g., mu, beta, and MRCP band). To our knowledge, this is the first work reporting comprehensive comparisons of all these influence factors on the lower-limb movement intention detection. Our decoding and comparison results can shed light on the design of a high accuracy and short latency brain switch for lower-limb rehabilitation applications.

## 2. Materials and methods

### 2.1. Experimental protocol and set-up

Ten healthy right-handed subjects (three females, average age 25.6 ± 2.9 years old) participated in the experiment. The experiment conformed to the Declaration of Helsinki and the protocol was approved by the local ethical committee (EPFL-Brain and Mind Institute ethical committee). All participants provided written informed consent for this study.

Each participant was measured during one single session, which consisted of 6 runs (around 12 min each) with 3 min rest in between. Participants were comfortably seated in a lower-limb gait trainer, named the legoPress (Olivier et al., 2014). The legoPress is a robotic device that can mobilize the user's legs driven by a BMI (Liu et al., 2017). It has integrated force sensors to monitor the interactions at the pedal level. We used two monitors in the recordings: the first one was placed approximately 2 m away to display the instructions (e.g., visual cues) to the subjects, and the other one was used to present the produced pressure from the movements with a graphical user interface (GUI) to the experimenter. The force was monitored in real time to keep the isometry of the movement in each trial and to eliminate corrupted trials.

**Figure 1:**
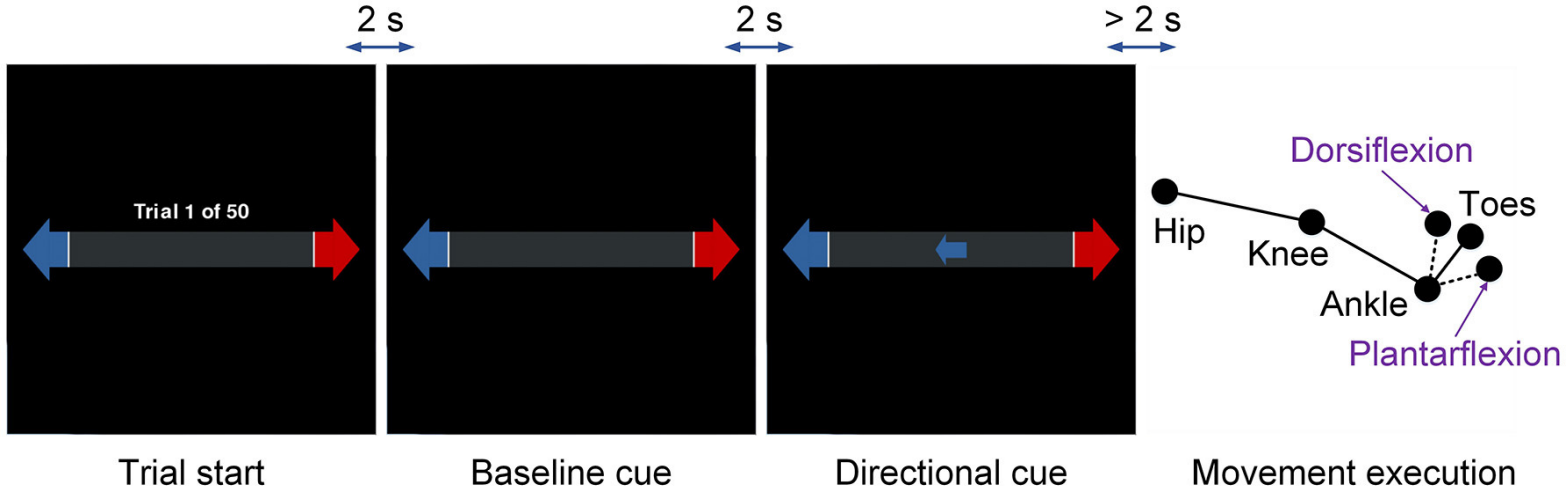
Protocol of the experiment. The movement type refers to ankle dorsiflexion and plantar flexion, and the limb side refers to left and right legs. The movement type was consistent within each run and the limb side was indicated by the directional cues.

The subject performed self-paced dorsiflexion for 3 runs and plantar flexion for the other 3 runs with a crossover design. Each run consisted of 50 trials with left and right directional cues randomized and balanced inside. Figure 1 presents the protocol of the experiment. Each trial began with a cue showing the number of performed trials, followed by an idle period (baseline) of 2 s and a preparation period of more than 2 s. The preparation period refers to the time interval after the directional cue but before the actual movement. The subject was instructed to wait at least 2 s to execute the movement after the directional cue, but with no explicit or implicit count of the time. Trials with the preparation period less than 2 s were removed to avoid possible contamination with instructional visual stimuli.

### 2.2. Data acquisition and preprocessing

EEG, EMG, and EOG signals were synchronously acquired using an ActiveTwo measurement system (BioSemi instrumentation, Amsterdam, Netherlands), with a sampling frequency of 2048 Hz. EEG was recorded using 32 electrodes arranged in the modified 10/20 international standard. Three EOG electrodes were placed above the nasion and below the outer canthi of the eyes to capture both horizontal and vertical EOG components. Besides, muscular activity was inspected by 4 EMG electrodes to detect the actual movement onset. In dorsiflexion runs, two pairs of surface EMG (sEMG) electrodes were placed on the tibialis anterior muscles of each leg. The sEMG electrodes were placed on the tibialis posterior muscles for plantar flexion runs. This configuration was shown to best capture the maximum voluntary contraction (MVC) of the muscles (Soma et al., 2013).

Data acquisition, visualization, and processing were conducted under a customized Python framework (Lee et al., 2017). Trigger values were synchronized with the electrophysiological signals via a USB to parallel port adapter from the legoPress. The directional cues were stored in local files and trials when the subject executed the movement incorrectly were removed in the post processing. The actual movement onset was detected from sEMG based on a data conditioning and threshold-based method (Solnik et al., 2010). The data conditioning included band-pass filtering at 30-300 Hz using a sixth order Butterworth filter, the Teager-Kaiser energy operator (TKEO), rectification and low-pass filtering at 50 Hz using a second order Butterworth filter. The TKEO was defined as,

(1) $$\begin{array}{r} \psi \left[x\left(n\right)\right]=x{\left(n\right)}^{2}-x\left(n-1\right)x\left(n+1\right) \end{array}$$

where *x* was the EMG signal and *n* was the sample index. This conditioning was shown to reduce high-frequency noise and provide a smoothed envelope of the signal (Solnik et al., 2010). After data conditioning, we calculated the threshold as

(2) $$\begin{array}{r} T=\mu +h\theta \end{array}$$

where μ and θ were the mean and standard deviation of the reference signal. The reference signal was chosen as [0, 1] s with respect to the directional cue since there were no sudden muscle activities during this period. *h* was a preset variable defining the level of the threshold. In this study, we empirically set *h* to 10. The actual movement onset was identified as the first time point when there were more than 50 consecutive samples exceeded the threshold *T*. We removed the trials in which no movement onset was detected by this method. Visual inspections were also performed to remove noisy trials.

As the EEG signals might be contaminated by the eye movement, we performed artifact removal by calculating the correlation between EOG and EEG channels. We extracted the idle periods (baseline) and used a regression-based method to evaluate the influence of both horizontal and vertical EOG component on each EEG channel (Lew et al., 2012). In contrast to eliminating peripheral channels (Zhang et al., 2015), we kept all the 32 channels in the preprocessing but only removed the EOG component.

### 2.3. Electrophysiology analysis

Grand average EEG analysis was first performed to visualize the brain activity. The EEG signals were spatially filtered by a common average reference (CAR) and a weighted average filter (WAVG), which had been proved to improve the detection performance of motor intention (Khaliliardali et al., 2015). A 4th order non-causal Butterworth filter with cutoff frequencies between [0.1, 1] Hz was applied to filter the data. While the grand average analysis was used to show the EEG scalp distributions, we extracted MRCPs and SMRs from the raw signals to assess the pre-movement cortical activity.

For MRCP analysis, we applied CAR to remove the global background activity. The signals were filtered using the same Butterworth filter (4th order, zero-phase, at [0.1, 1] Hz) and then down-sampled to 16 Hz. The EEG data were segmented into 8 s long epochs from −6 to 2 s with respect to the actual movement onset. Each epoch was baseline corrected with the average activity between [1, 2] s with respect to the baseline cue.

On the other hand, for SMR analysis, the EEG data were first spatially filtered by a small Laplacian, i.e., the signal of each channel was referenced to the averaged potentials from the nearest orthogonal electrodes. Then the data were down-sampled to 512 Hz. The time-frequency representation of the power was calculated using Morlet wavelets with 5 cycles in the frequency band of 0.1–30 Hz (López-Larraz et al., 2014; Sburlea et al., 2015). Each trial contained samples from −6 to 2 s with respect to the movement onset. Bootstrap statistics were computed using a spectral baseline window from [−4, −3] s with respect to the actual movement onset at a significance level of α = 0.05, and non-significant values were zeroed out.

### 2.4. Feature extraction and classification

The pipeline from preprocessing, feature extraction, and classification were performed on each movement type and limb side. For both EEG correlates, the features were calculated over 1 s windows shifted with the step of 125 ms on the 8 central channels (FC1, C3, CP1, Pz, CP2, FC2, Fz, and Cz). The EEG traces in the time domain were used as features for the MRCP-based method. In contrast, we calculated the power spectral density (PSD) using a Welch's method as the features for the SMR-based method (pwelch function implemented in Matlab). The Welch's method first segmented the time series into different intervals. A modified periodogram was computed for thses segments and the resulted values were averaged to produce the estimate of the PSD. The PSD was estimated using Hamming windows of 0.75 s overlapped of 50%, and then log-transformed to fit the normality.

For feature extraction, we applied the canonical variant analysis (CVA), also known as multivariate discriminant analysis, which had shown to be advantageous for BMI (Galán et al., 2007). The intuition of CVA is to extract canonical discriminant spatial patterns (CDSPs) whose directions maximize the separability in the features between the given classes. By extracting CDSPs from the original feature space, CVA ranked the discriminant power (DP) based on the differences between the given classification tasks. The features were extracted from preprocessed EEG signals with different units. For the SMR-based method, the features were PSD values (uV^2^/Hz) with channel and frequency band pairs; While for the MRCP-based method, the features were EEG traces (uV) with channel and time point pairs.

The amplitudes or power estimations obtained from −1.5 to 0 s with respect to the movement onset were labeled as preparation epochs and the others (from −6 to −1.5 s and from 0 to 2 s) were labeled as idle epochs. The 10 most discriminative features ranked by CVA were selected for classification. We used linear discriminant analysis (LDA) to classify preparation and idle epochs (classify function implemented in Matlab). We assumed that the two classes had the same corvarience matrixes and the prediction was performed based on Bayes theorem. The sample-based classification was conducted using a 5-fold cross validation, where the chronological order of the data was maintained. Since the number of idle and preparation epochs was imbalanced, we further reported the results with an area under the curve (AUC) in the receiver operating characteristics (ROC) space, which represented the trade-off between the false positive rates (FPR) and true positive rates (TPR). It is worth noting that the CVA was only performed on the training data in each fold.

Furthermore, we performed single-trial classification to evaluate the latency. The amplitudes or power estimations computed exactly at the actual movement onset (with the same 1-s window from −1 to 0 s) were labeled as the preparation period and the estimations at directional cue were labeled as the idle period. We used the same CVA and LDA for feature selection and classification over 5-fold cross validation. To evaluate the single-trial performance, we used 1 s window shift every 125 ms from −4 to 2 s with respect to the actual movement onset. The chance level was computed by shuffling the training labels and repeated the 5-fold cross validation for 1,000 times. When 3 consecutive samples had a true positive rate significantly above chance level (two-sample *t*-test, *p* < 0.05, Bonferroni correction), we selected the first time point as the detection latency.

### 2.5. *Post-hoc* feature and influence factor analysis

We did a post-hoc analysis to assess the relevant features for classification and the impact of the influence factors on the performance. We first measured the discriminability between the preparation period and idle period. A modified CVA was used to calculate the discriminant power (DP) of the two classes. Given the feature number *c*, class number *k* and feature matrix *T* (with the dimension of *c* × (*k* − 1)), the discriminant power of a single feature can be calculated as,

(3) $$\begin{array}{r} DP_{e}=100\times \frac{\sum_{i=1}^{N}\sum_{u=1}^{k-1}\gamma_{u}^{i}{t_{eu}^{i}}^{2}}{\sum_{i=1}^{N}\sum_{e=1}^{c}\sum_{u=1}^{k-1}\gamma_{u}^{i}{t_{eu}^{i}}^{2}} \end{array}$$

where *N* was the number of data chunks across different influence factors, *t*_*eu*_ was the element of *T*, and γ_*u*_ was the normalized eigenvalues of feature matrix defined as,

(4) $$\begin{array}{r} \gamma_{u}=\frac{\lambda_{u}}{\sum_{u=1}^{k-1}\lambda_{u}} \end{array}$$

where λ was the eigenvalues of the CDSP matrix. Compared with averaging DP values across all the data chucks, the modified CVA was able to better capture the variations among different influence factors. It also penalized those features which were not consistently discriminant. Furthermore, DP distributions were estimated by averaging the discriminant power across the frequency bands and time points for the SMR-based and MRCP-based method, respectively.

The four influence factors can be categorized into experimental conditions (movement types and limb sides) and processing methods (MRCP-based or SMR-based framework with different sub-frequencies). In order to evaluate the performance with different frequency bands, we conducted the movement onset detection with the signals filtered in certain frequency intervals, i.e., MRCP ([0.1, 1] Hz), delta ([1, 4] Hz), theta ([4, 7] Hz), mu ([8, 13] Hz), low beta ([14, 21] Hz), and high beta ([22, 30] Hz) bands. In contrast to manually setting the feature number to 10, a grid search was used to optimize the feature number during feature selection. Finally, we built a concatenate model with the combination of MRCP-based and SMR-based features and used this model to estimate both the classification performance across all the six frequency bands and the detection latency in a single-trial basis.

## 3. Results

### 3.1. Electrophysiology analysis

**Figure 2:**
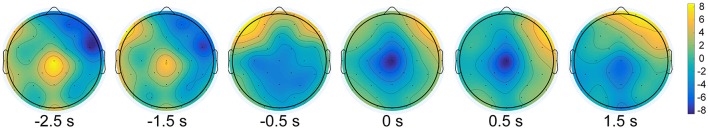
Topographic representations of the brain activity at different time points from −2.5 s to 1.5 s with respect to the actual movement onset.

**Figure 3:**
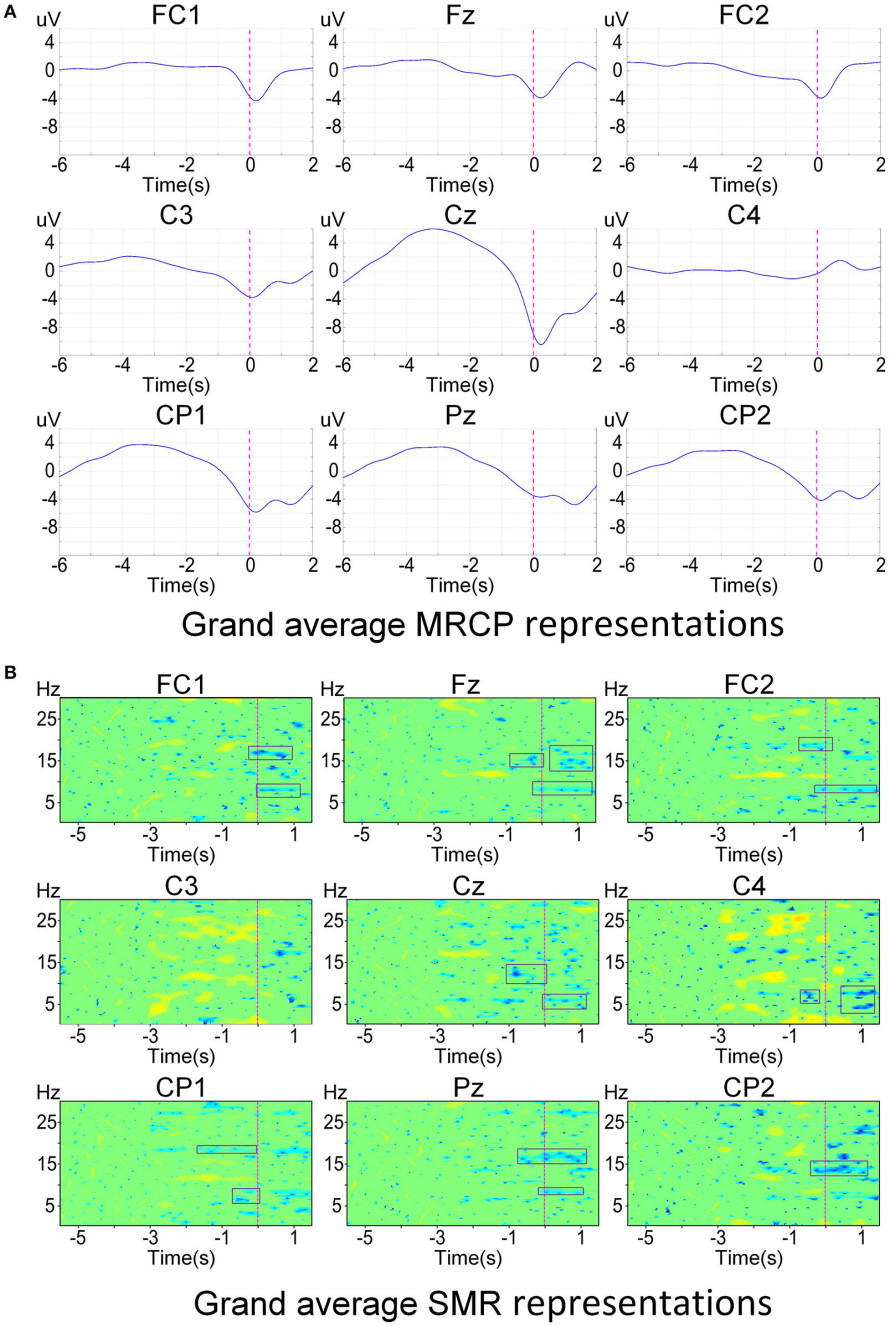
Grand average electrophysiology signals around the motor cortex over all subjects and conditions. The actual movement onset is shown with vertical dashed lines. **(A)** Grand average time-amplitude representations at [0.1, 1] Hz. **(B)** Grand average time-frequency representations at [0.1, 30] Hz. Only significant values (bootstrap *p* < 0.05) are colored and non-significant values are plotted in green.

Figure 2 shows the brain activity at different time points averaged over both subjects (*N* = 10) and conditions (movement types and limb sides) across all 32 channels. We observed that the negativity was spatially localized in the central area and started around −1.5 s with respect to the actual movement onset. The negative potentials were prominent elicited over regions involved in motor planning and motor execution. Furthermore, grand average EEG correlates, i.e., MRCPs and SMRs, are shown in Figure 3. The electrophysiology results observed in this study are consistent with previous literature on both upper-limb (López-Larraz et al., 2014) and lower-limb works (Sburlea et al., 2015). Statistical test on the EEG signals from Cz found that there was a significant difference (two-sample *t*-test, *p* < 0.05) between the movement types, while no significant difference (two-sample *t*-test, *p* > 0.05) was observed between the limb sides.

### 3.2. Classification results

Both the sample-based and single-trial classification were performed separately across the influence factors. Since the MRCP has been proved prominent in the frequency band of [0.1,1] Hz (Lew et al., 2012; Garipelli et al., 2013), we only exploited this narrow band in the MRCP-based method. The performance of SMR-based method across sub-frequency bands will be discussed in the following section. The remaining influence factors include the movement type, limb side, and processing method. An average area under the curve (AUC) of 91.0 ± 3.5% and 64.7 ± 4.3% was obtained for the MRCP-based and SMR-based method, respectively. No statistically significant difference (two-sample *t*-test, *p* > 0.05, Bonferroni correction) was found between left and right legs, although the performance of left leg was slighter better than right leg with the *p*-value close to the significance level (*p* = 0.087). We pooled the trials with left and right legs for sample-based classification. It is worth noting that we also performed the MRCP-based processing for detecting movement onset with different frequency bands. The classification results were close to chance level (Müller-Putz et al., 2008) and therefore is not reported.

**Figure 4:**
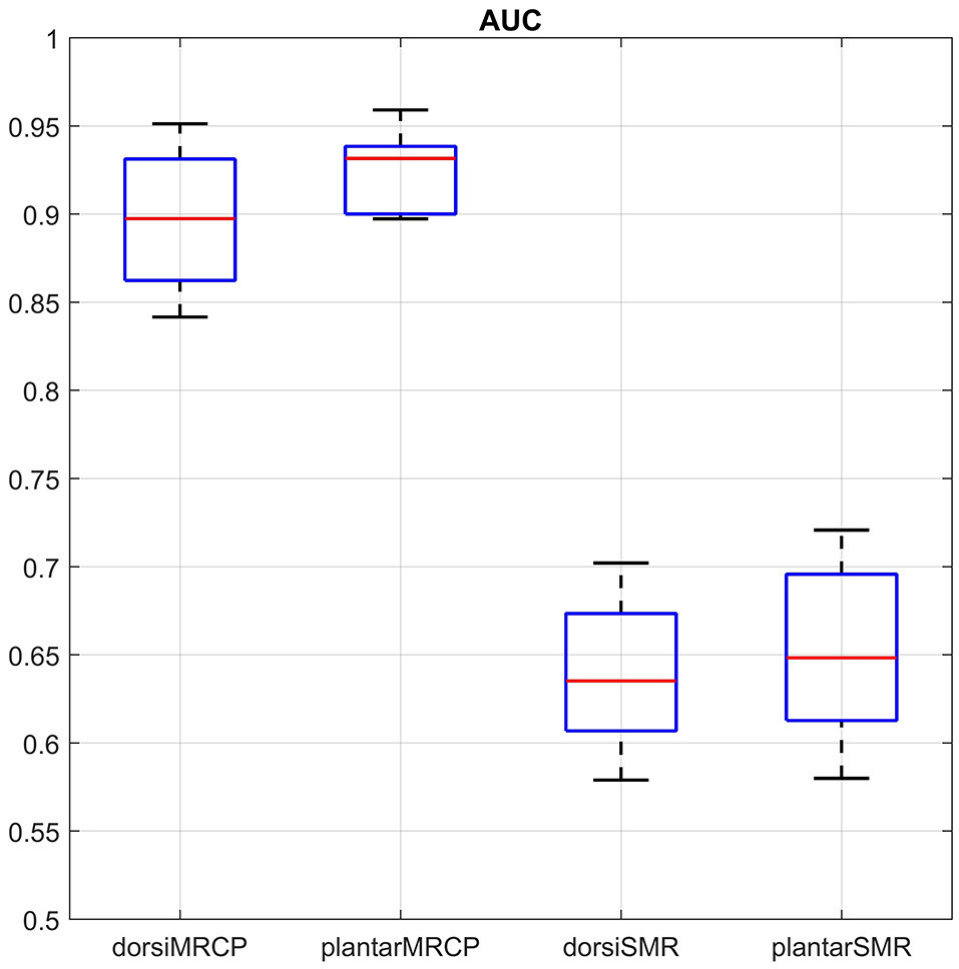
Sample-based performance (AUC) for different movement types and processing methods. We use dorsi and plantar to represent dorsiflexion and plantar flexion for simplicity. The boxplot shows the median (central mark) and 25th/75th percentiles (edges of the box) of the AUC values.

Figure 4 shows the AUC concerning the movement types and processing methods. Plantar flexion with the MRCP-based method reached the highest AUC (92.5 ± 2.2%). A repeated two-way ANOVA on AUC found no significant differences between movement types (*p* = 0.131) and no significant interaction (*p* = 0.422) between these two factors. However, a significant difference (*p* < 0.01) was observed between processing methods with the MRCP-based method performed better. Besides, the plantar flexion with the MRCP-based method also reached the lowest error rate (14.7 ± 2.7%). Another repeated two-way ANOVA was performed on the error rate with similar results (*p* = 0.080, 0.667 and < 0.01, respectively).

**Figure 5:**
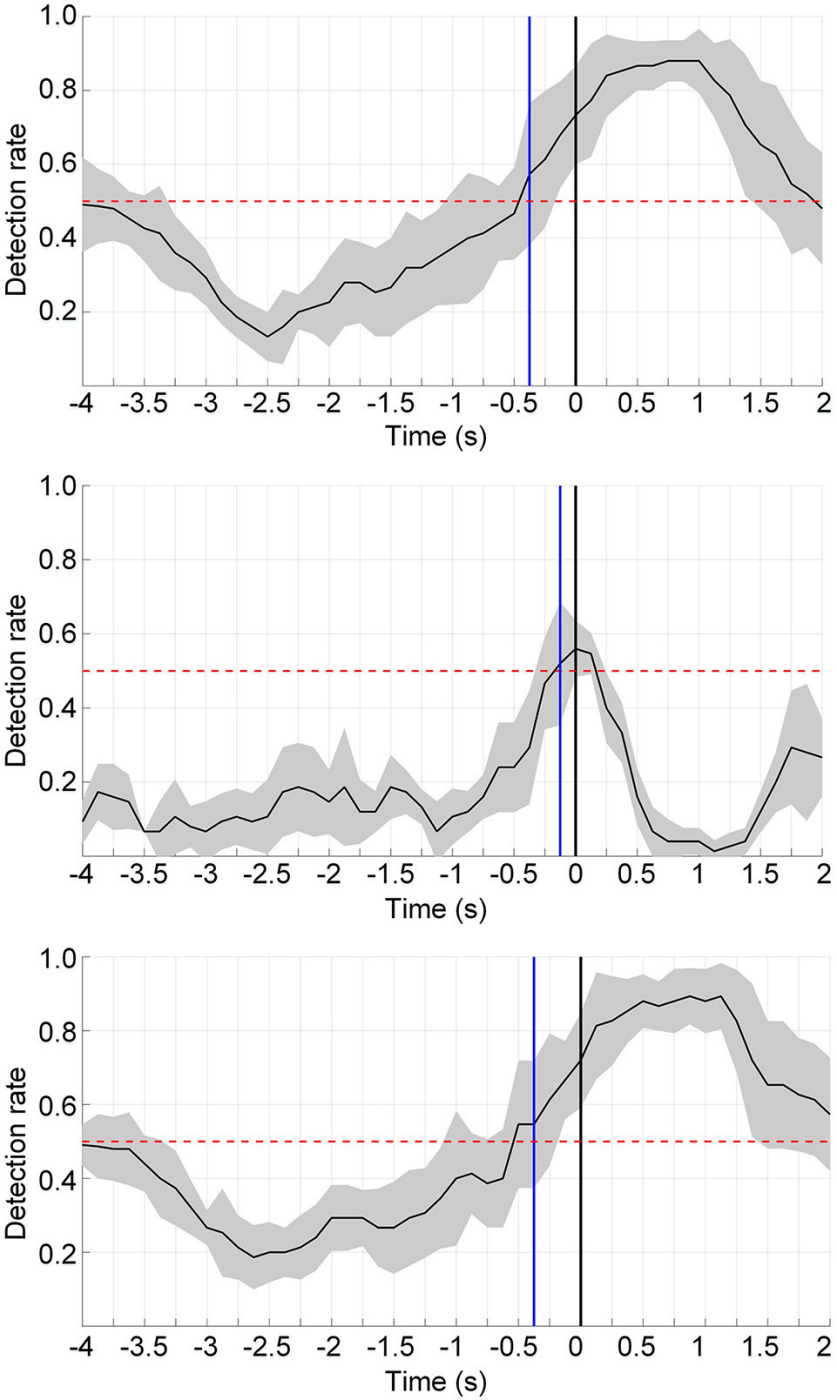
Single-trial detection performance using the MRCP-based method **(upper)**, the SMR-based approach **(middle)**, and the concatenate model combining MRCPs and SMRs **(lower)** from a typical subject (s10). The detection was performed from −4 to 2 s with respect to the actual movement onset. The blue lines display the detection points when consecutive 3 samples have a detection rate significantly above the chance level (*p* < 0.05). The chance level is shown in red dashed lines. The black curves and gray regions depict the mean and standard deviation of the detection rate.

Single-trial classification of the movement intention detection was performed across the three influence factors: the movement type, limb side, and processing method. The results of one representative subject for the two processing framework are shown in Figure 5 (upper and middle panels). The black curves display the mean detection rate across the 5 folds with the standard deviation in gray shadows. The results of the latency (detection time) over all subjects with these influence factors are displayed in Table 1. The average detection latencies for all subjects across all influence factors were −0.334 ± 0.216 s, ranging from −1 to 0.25 s with respect to the movement onset. A repeated three-way ANOVA on the latency found no three-way interaction (*p* = 0.951) and no two-factor interactions (*p* = 0.426, 0.951, 0.668). No significant difference (two-sample *t*-test, *p* > 0.05, Bonferroni correction) was observed between the latencies of the processing method. We also observed that the majority of the MRCP-based detection occurred at around 300 ms before the actual movement onset, which was in agreement with previous works (Lew et al., 2012; Ibáńez et al., 2014). For the SMR-based detection, the average latency was similar to the MRCP-based results, whereas there were several detections occurring after the movement onset, which might be caused by the false negatives during the movement preparation periods. Furthermore, Table 2 displays the single-trial detection performance with the concatenate model using both MRCP and SMR features. The result of one representative subject for this processing framework is shown in Figure 5 (lower panel). We observed that similar results were obtained between the MRCP-based method and the concatenate approach, indicating that MRCPs might be more frequently selected as the discriminative features in the single-trial detection.

**Table 1:** Single-trial detection performance (latency) for each subject with different influence factors.

**Subject ID**	**MRCP-based method**				**SMR-based method**			
	**dorsiL**	**dorsiR**	**plantarL**	**plantarR**	**dorsiL**	**dorsiR**	**plantarL**	**plantarR**
s1	−0.125	−0.250	−0.250	−0.125	−0.250	−0.500	−0.125	−0.375
s2	−0.250	−0.375	−0.125	−0.375	−0.250	+0.125	−0.250	−0.625
s3	−0.750	−0.500	−0.500	−0.500	−0.250	−0.875	+0.125	−0.125
s4	−0.375	−0.375	−0.750	−0.750	−0.500	−0.375	−0.750	−1.000
s5	−0.375	−0.625	−0.250	−0.875	−0.500	+0.125	−0.750	−0.375
s6	−0.250	−0.375	−0.375	−0.375	−0.500	−0.500	+0.250	−0.125
s7	−0.375	−0.375	−0.500	−0.500	−0.125	−0.750	−0.500	−0.250
s8	−0.250	−0.375	−0.375	−0.500	−0.375	−0.500	−0.250	−0.375
s9	−0.250	−0.500	−0.375	−0.375	−0.125	−0.500	−0.125	−0.250
s10	−0.375	−0.375	−0.250	−0.375	−0.250	−0.375	−0.500	−0.125
Mean ± Std	−0.338 ± 0.167	−0.413 ± 0.103	−0.375 ± 0.177	−0.475 ± 0.211	−0.313 ± 0.147	−0.413 ± 0.323	−0.288 ± 0.339	−0.363 ± 0.273

*The unit is second with respect to the actual movement onset*.

**Table 2:** Single-trial detection performance (latency) for each subject with the combination of MRCPs and SMRs.

**SubjectID**	**dorsiL**	**dorsiR**	**plantarL**	**plantarR**
s1	−0.125	−0.250	−0.125	−0.250
s2	−0.250	−0.250	−0.125	−0.125
s3	−0.500	−0.500	−0.375	−0.375
s4	−0.375	−0.375	−0.500	−0.500
s5	−0.375	−0.500	−0.250	−0.500
s6	−0.250	−0.375	−0.375	−0.250
s7	−0.250	−0.500	−0.500	−0.250
s8	−0.250	−0.375	−0.250	−0.375
s9	−0.250	−0.500	−0.250	−0.250
s10	−0.375	−0.375	−0.375	−0.375
Mean ±	−0.300 ± 0.105	−0.400 ± 0.099	−0.313 ± 0.135	−0.325 ± 0.121
Std				

*The unit is second with respect to the actual movement onset*.

### 3.3. Feature and influence factor analysis

**Figure 6:**
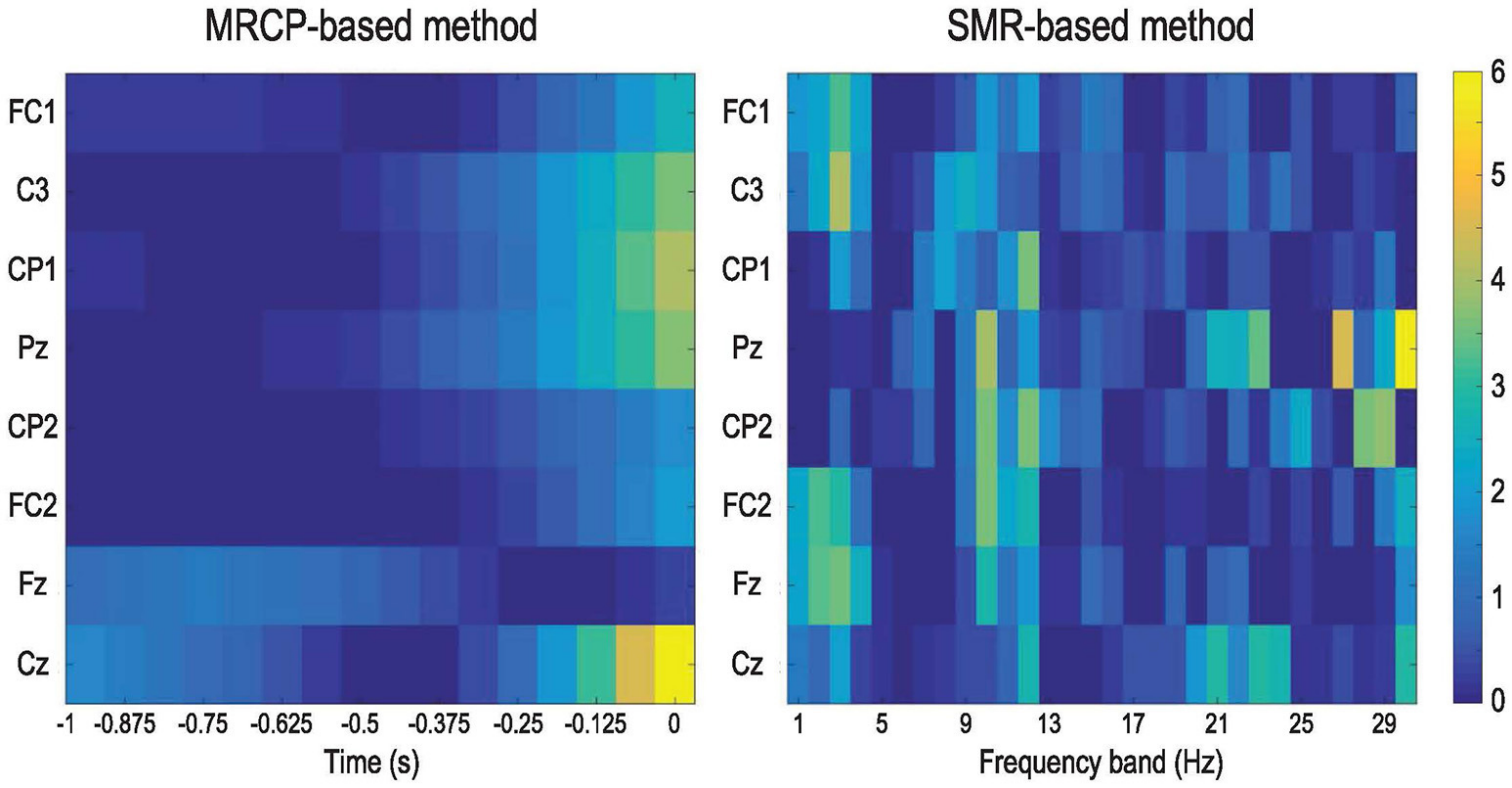
DP maps calculated by the modified CVA to show the consistency in feature selection across all subjects and conditions. The features are the channels and time points in the 1-s window for the MRCP-based method and channels and frequency bands for the SMR-based method.

**Figure 7:**
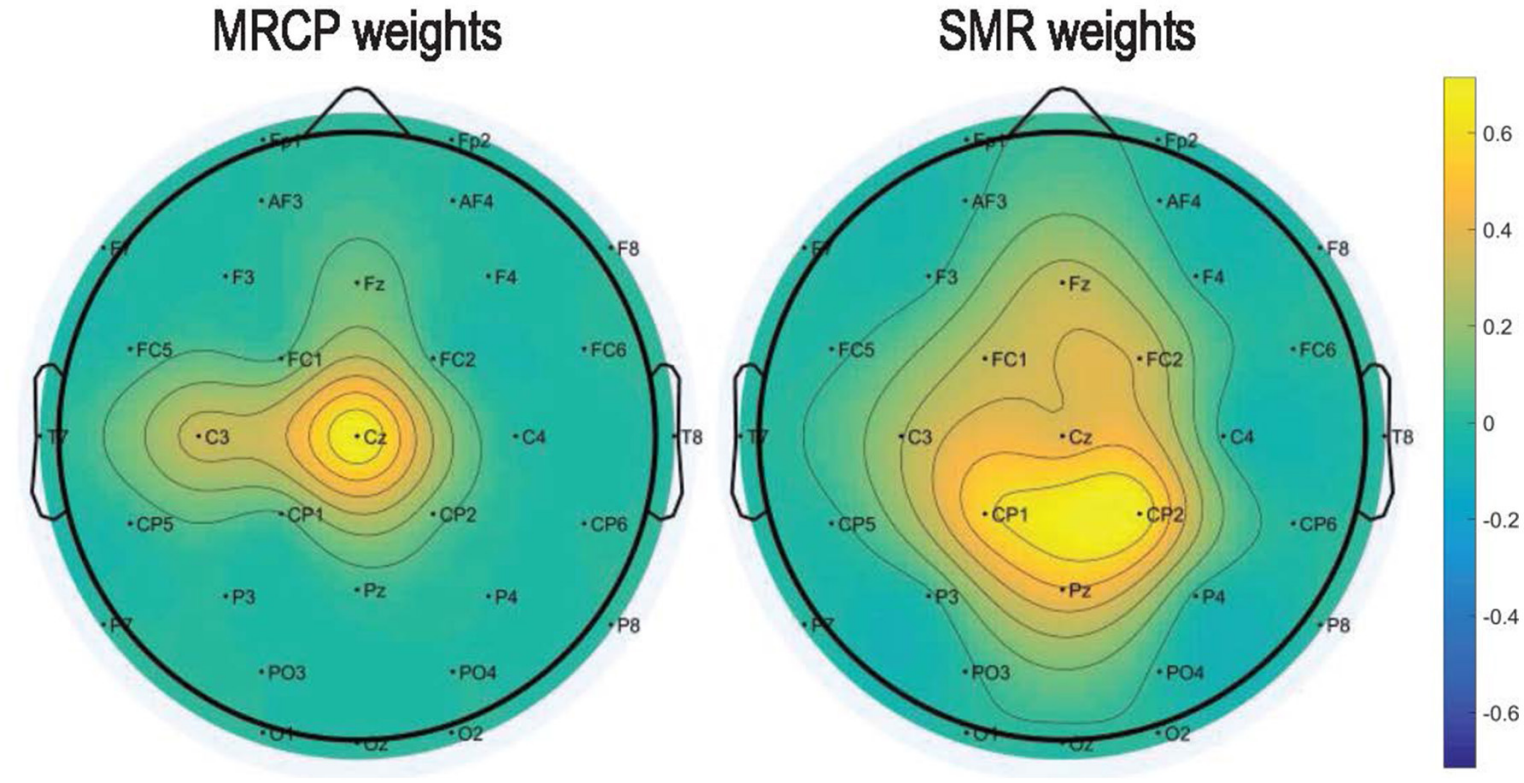
Topographic maps to show the normalized DP index of each channel averaged across all subjects and conditions. The weights were assigned by the modified CVA for the MRCP-based and SMR-based methods.

Figure 6 presents the DP maps attained by both processing methods using the modified CVA. For the MRCP-based method, central channel Cz in the last two windows before movement onset were the most discriminative features. Besides, CP1, Pz, and C3 in the last windows before actual movement were also discriminative between the preparation and idle periods. For the SMR-based method, the most discriminative features distributed at the high beta band at parietal midline vertex. Bins in the frequencies [9, 12] Hz (mu band) and [1, 4] Hz (delta band) were also selected in various channels. Based on the DP maps, it seems that the MRCP-based and SMR-based processing methods carried complementary information. Scalp distributions of the discriminate power are shown in Figure 7. Each topographic map shows the weights assigned by the modified CVA. We observed that the central areas of the motor cortex were the regions with the highest weight for the MRCP-based method. For the SMR-based method, a wide distribution was found along the motor cortex, indicating the subject-specific brain modulations.

**Figure 8:**
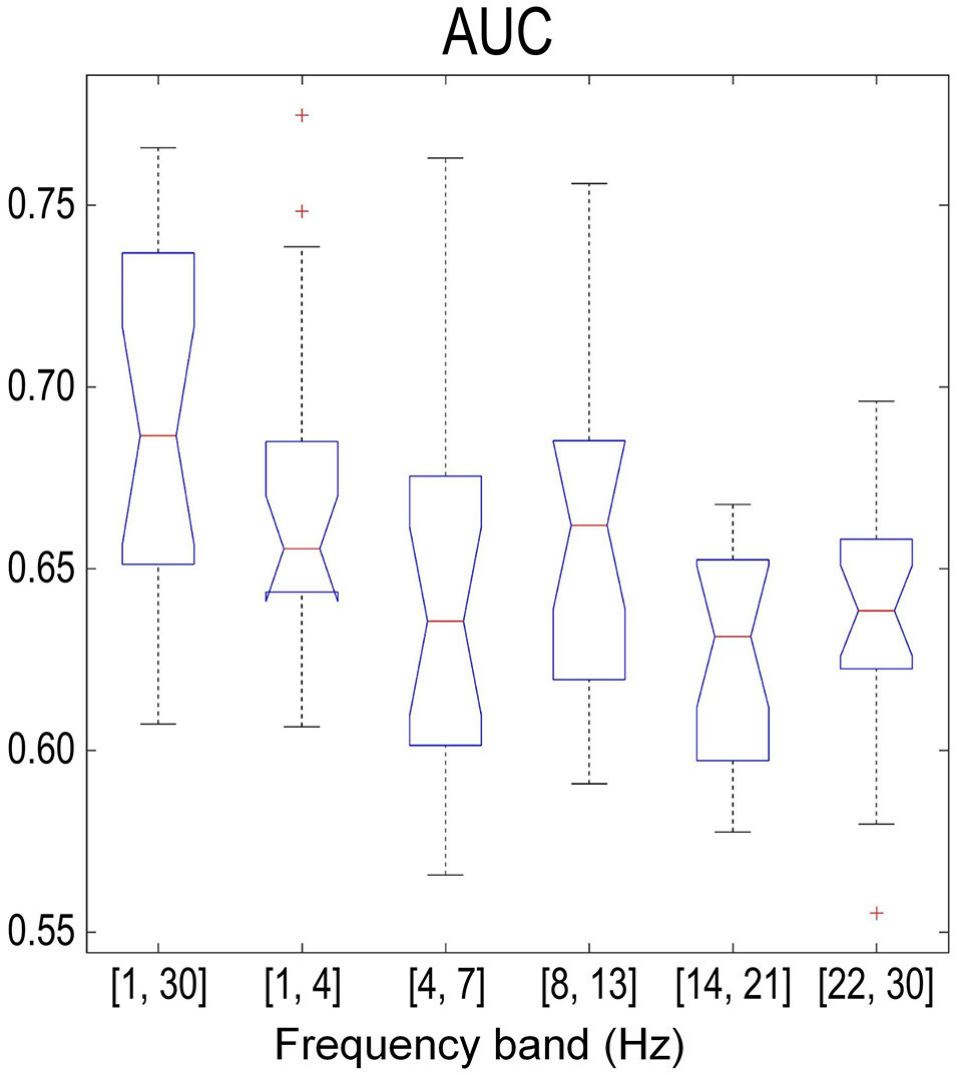
Sample-based performance (AUC) across different frequency bands using the SMR-based processing method. The meaning of the boxplot was the same as that in Figure 4.

**Figure 9:**
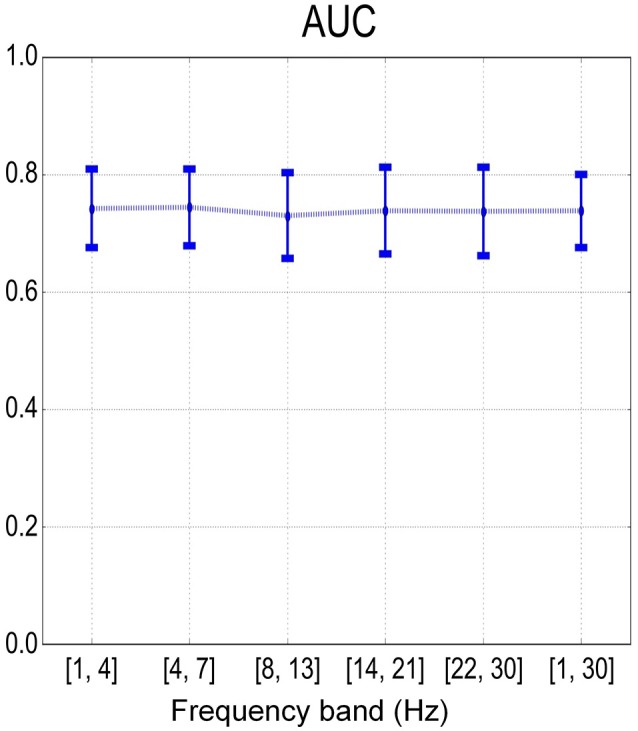
Mean and standard deviation of sample-based performance (AUC) across different frequency bands using the combination of MRCP and SMR features.

Figure 8 summarizes the sample-based performance for the SMR-based method across different frequency bands. We found a significant difference (one-way repeated measures ANOVA with factor frequency band, *p* < 0.01) between frequency bands. Multiple comparisons with the Tukey-Kramer critical value showed that the mean AUC of the full band (68.2 ± 4.6%) was significantly larger than the mean AUC from other frequency bands. Similar results were also found with one-way repeated measures ANOVA and multiple comparisons on the error rate over the factor of frequency bands. Besides, the detection results based on the combination of MRCP-based and SMR-based methods are shown in Figure 9. No significant difference was found (one-way repeated measures ANOVA with factor frequency band, *p* > 0.05) in both AUC and error rate between frequency bands. Finally, the performance of the combination of features was better than the SMR-based method but worse than the MRCP-based method (one-way repeated measures ANOVA, *p* < 0.01, and multiple comparisons with the TukeyKramer critical value), which was consistent with previous works with both upper-limb and lower-limb movement intention detection (Ibáńez et al., 2014; Sburlea et al., 2015).

## 4. Discussion

In this paper, we studied the problem of detecting a single lower-limb movement from EEG signals comparing four influence factors on the performance, i.e., the limb side, movement type, processing framework and frequency band. We found that plantar flexion with left leg using the MRCP-based method in [0.1, 1] Hz was the optimal combination in terms of classification performance, i.e., AUC. The performance of the MRCP-based method in a single-trial basis was significantly better than that of the SMR-based method. For both processing methods, the results from feature analysis were consistent with the electrophysiological results. We also observed that a combination of both features could boost the detection performance. The overall detection and comparison with the influence factors in this study could shed light on the design of more practical BMI systems.

In recent years, there have been an increasing number of studies investigating the decoding of movement onset with MRCP-based techniques (Bhagat et al., 2016; Pereira et al., 2017), SMR-based approaches (Bai et al., 2011; Planelles et al., 2014), and the concatenated methods (Ibáńez et al., 2014; Sburlea et al., 2015). We observed from our study that the EEG correlates of lower-limb movement intention shared common features with those of upper limbs. The practical significance of the current work in a translational perspective is the single trial classification of the movement intention detection. We showed the applicability of the method in people that have plastic changes of their brain and brain responses because of the neurological disease.

Commonly used metrics to estimate the performance of onset detection include the true positive rate (TPR), false positive/min, and detection latency (Ibáńez et al., 2014; Xu et al., 2014a; Lin et al., 2016). As the main goal of this work is to estimate the influence factors on the performance, we only use the detection latency in a single-trial basis and AUC for sample-based classification as our metrics. We obtained an average detection latency of −0.334 ± 0.216 s across all conditions and the AUC of 91.0 ± 3.5% and 64.7 ± 4.3% for MRCP and SMR detection, respectively. Comparable works can be found in Bai et al. where the latency was 0.62 s and the successful prediction rate was 90% (Bai et al., 2011), Lew et al. where the latency was −0.460 ± 0.085 s and the classification performance was 92% (Lew et al., 2012), and Khaliliardali et al. where the latency was −0.320 ± 0.200 s and the classification performance was 83.0 ± 13.0% (Khaliliardali et al., 2015).

The influence factors proposed by the current work have been partially studied in previous works. For example, analysis of frequency band in the upper-limb movement intention detection was conducted in Garipelli et al. (2013) and Lew et al. (2012) with a cue-based (CNV) and self-paced (BP) paradigm, respectively. Different processing methods were compared in Sburlea et al. (2015), i.e., MRCP-based, SMR-based, concatenated of both features, and the combination of both model outputs. No significant difference was also found concerning the limb side, i.e., left or right foot, in that paper, which was consistent with the current study. Although recent works have also compared influence factors during movement imagination (Xu et al., 2015), in this study we have focused on real movements because our aim is to integrate a brain switch in a robotics rehabilitation framework, where patients have to attempt the movement rather than just imagine it. Another potential influence factor concerning motor execution is the type of the paradigm, e.g., cue-based (reaction tasks) or self-paced. Previous work on sitting and standing intention decoding found that there was no significant difference in classification accuracy between these two paradigms (Bulea et al., 2014).

In order to build a practical brain switch in a closed-loop BMI, detection of movement intention with a short latency was a prerequisite. Causality of the spectral filter is the main issue when transferring from offline to online works. A drop of TPR would be expected in the online decoding with MRCP-based methods (Niazi et al., 2011; Xu et al., 2014a). In the current work, although the MRCP-based method was shown to be superior in classification performance, the SMR-based method might be more suitable for online deployment. A direct comparison of the results from this study with previous works is difficult due to the different settings and performance metrics. Still, results of the electrophysiology and feature analysis in the current work were consistent with the literature (Bai et al., 2011; Lew et al., 2012; Bulea et al., 2014; Sburlea et al., 2015). The comparison of impact factors presented in this study could serve as a guidance for the design of a practical BMI. Future work would be devoted to building this brain switch in a closed loop, with visual or proprioceptive feedback provided during an online experiment to trigger the gait trainer. Similar work had been conducted by Bhagat et al. where successful intent detection was used to trigger an upper-limb exoskeleton (Bhagat et al., 2016). Moreover, our results on healthy subjects need to be validated on patients as the clinical target. Previous studies had found that similar results were obtained in movement intention detection from both healthy subjects and patients (López-Larraz et al., 2014; Xu et al., 2014c), although medication status could be another influence factor in the clinical environment. Further studies with patients should be carried out toward the gait rehabilitation in clinical trials.

## Author contributions

DL, WC, and JM were responsible for the study conception; DL ran the experiments and collected the data; DL, WC, RC, ZP, and JM contributed to the methodology, data analysis, and manuscript preparation. All authors read and approved the final manuscript.

### Conflict of interest statement

The authors declare that the research was conducted in the absence of any commercial or financial relationships that could be construed as a potential conflict of interest.
